# Feeding and Nutritional Key Features of Crisponi/Cold-Induced Sweating Syndrome

**DOI:** 10.3390/genes15091109

**Published:** 2024-08-23

**Authors:** Roberta Onesimo, Elisabetta Sforza, Federica Palermo, Valentina Giorgio, Chiara Leoni, Donato Rigante, Valentina Trevisan, Cristiana Agazzi, Domenico Limongelli, Francesco Proli, Eliza Maria Kuczynska, Laura Crisponi, Giangiorgio Crisponi, Giuseppe Zampino

**Affiliations:** 1 Centre for Rare Diseases and Transition, Department of Woman and Child Health and Public Health, Fondazione Policlinico Universitario A. Gemelli IRCCS, 00168 Rome, Italy; roberta.onesimo@policlinicogemelli.it (R.O.); giuseppe.zampino@policlinicogemelli.it (G.Z.); 2 Department of Life Sciences and Public Health, Faculty of Medicine and Surgery, Catholic University of Sacred Heart, 00168 Rome, Italy; 3 Institute for Genetic and Biomedical Research (IRGB), The National Research Council (CNR), Monserrato, 09042 Cagliari, Italy; 4 Independent Researcher, 09042 Cagliari, Italy; crisponi@gmail.com

**Keywords:** CRLF1, rare disease, dysphagia, precision medicine, genetics

## Abstract

Feeding difficulties are constantly present in patients with Crisponi/cold-induced sweating syndrome type 1 (CS/CISS1). The aim of our study was to describe their prevalence and evolution from birth to adult age. We performed an observational study at the Department of Life Sciences and Public Health, Rome. Fourteen patients were included in this study (six M; mean age: 18 years; SD: 10.62 years; median age: 15 years; age range: 6–44 years); six were adults (43%). Data on oral motor abilities from birth were collected. Meal duration, presence of swallowing reflex, dysphagia symptoms, difficulty chewing, and drooling management were assessed. At birth, all patients needed enteral feeding. Introduction of solid food was postponed beyond the age of 18 months in 43% of patients. During childhood and adolescence, mealtime was characterized by increased duration (43%) accompanied by fatigue during chewing (43%), food spillage from the nasal cavities (21%), sialorrhea (86%), and poor/reduced appetite (57%). A mature rotatory chewing skill was never achieved. This report expands the phenotype description of CS/CISS1 and also improves the overall management and prevention of complications in this ultra-rare disease.

## 1. Introduction

Crisponi/cold-induced sweating syndrome type 1 (CS/CISS1, OMIM #272430) is a severe ultra-rare genetic disease with autosomal recessive inheritance, described for the first time in 1996 by Giangiorgio Crisponi [1]. There are currently approximately 100 individuals with CS/CISS1 with mutations in the *CRLF1* gene [2,3], mostly European in ancestry. Interestingly, the highest concentration is recorded in Sardinia (Italy), where an incidence of 1 case per 20,700 new births is observed [4].

Due to considerable phenotypic overlap, the differential diagnosis should mainly consider two conditions: cold-induced sweating syndrome type 2 (CISS2, OMIM # 610313), caused by mutations in the *CLCF1* gene; and Stüve–Wiedemann syndrome/Schwartz-Jampel type 2 syndrome (SWS/SJS2, OMIM #601559), caused by mutations in the *LIFR* gene [5,6].

The “CRLF1/CLCF1” complex acts on cells expressing the ciliary neurotrophic factor receptor (CNTFR), and, upon binding, it activates the JAK/STAT signaling pathway [7]. This complex regulates the cell development and differentiation of various biological systems, such as the nervous, skeletal, hematopoietic, and immune systems [4]. 

CS/CISS1 is characterized by camptodactyly, tendency towards hyperthermia, and respiratory and/or feeding difficulties that can lead to sudden death in the first months of life [1,2].

Infants who survive over the first critical period gradually develop kyphoscoliosis, elevated plasma noradrenaline (NA) levels, and cold-induced sweating in their later years. In fact, this condition is characterized by profuse sweating induced not only by temperatures below 18–22 °C (paradoxical sweating) but also by strong emotional stimuli or ingestion of sweets [4]. Striking facial features include a large face, puckering lips, chubby cheeks, broad nose with anteverted nares, and long philtrum [1].

Although feeding problems have already been reported in CS/CISS1, most of these works are not based on standardized tests, and the evolution of the natural history of nutrition has not yet been reported.

This study aims, for the first time, to describe prospectively and to assess, with replicable tests, the level of feeding and swallowing abilities in patients with CS/CISS1 at different ages, from birth to adulthood; in addition, it specifically intends to quantify the development of feeding skills, helping to advance our comprehension of the natural history of this disorder.

## 2. Methods

### 2.1. Participants

Patients with a confirmed molecular diagnosis of CS/CISS1 syndrome were prospectively recruited over a six-month period at the Department of Life Sciences and Public Health, Fondazione Policlinico Agostino Gemelli-IRCCS, Rome, Italy. All these patients were previously reported [2,4,8,9,10,11]. No age restrictions were set. The patients were included in the study after signed informed consents were secured.

Fourteen patients were enrolled in this study (six M; mean age: 18 years; SD: 10.62 years; median age: 15 years; age range: 6–44 years); six were adults (43%). None of the included participants were siblings to other participants.

All patients were of Italian ancestry, and in most cases, they were Sardinian (*n* = 10/14, 71%). All study procedures were in line with the Declaration of Helsinki. The Local Ethical Committee (approval n.5518 of 2023) approved the study as part of an extensive protocol evaluation on disability in complex rare diseases.

All subjects carried a *CRLF1* mutation, either in homozygous or compound heterozygous state. The pathogenic variant c.676_677insA (p.T226NfsX104) in exon 4 was detected in nine out of fourteen patients (64%) in our cohort.

Among the striking clinical features, chubby cheeks and micrognathia were found in thirteen (93%) and seven patients (50%), respectively. Percentages of body and head features are reported in Table 1.

### 2.2. Procedure

As a first step, a pediatrician with expertise in rare diseases and complex disabilities collected a comprehensive familiar history. Patient demographics, genetics, and data regarding achieving key developmental milestones in feeding were systematically noted.

The clinical evaluation comprised a thorough physical examination. An accurate dental examination was also carried out to assess the possible presence of carious lesions.

A speech–language pathologist (SLP) directly observed each mealtime, following the best practice recommendation for oropharyngeal dysphagia [12]. The observation was aimed to provide contextual evidence of the nature and severity of feeding difficulties. Specifically, the observation focused on meal duration, presence of swallowing reflex, aspiration-related symptoms, difficulty in chewing, and secretion management [12]. 

Formal dysphagia screening tests were also administered. The tests were administered on the same day as the paediatric and SPL clinical assessment. Specifically, the Italian version of the Eating Assessment Tool-10 (I-EAT-10) for adult patients [13] and the Italian version of the Pediatric Eating Assessment Tool (I-Pedi-EAT-10) for pediatric patients [14,15] were administered to screen for the presence of swallowing disorders. The I-Pedi-EAT-10 responders were the primary caregivers. The items of both tools cover five different main domains: weight gain deficiencies; interference of the swallowing problems with the ability to go out for meals; the presence of excessive swallowing effort; the presence of stress and pain during eating and drinking; and food refusal and gag episodes during feeding. Responses are scored using a 5-point ordinal scale from “0” (no problem) to “4” (severe problem). Both scales are composed of 10 items [9,10,11]. A total score of 4 or more is considered abnormal, and a total score of 13 or more demonstrates high sensitivity and specificity in predicting penetration and/or aspiration issues [13,16]. The Karaduman Chewing Performance Scale (KCPS) was used to assess children’s chewing performance. The KCPS classifies chewing from ordinary to severely impaired within five levels of severity (0–4). Specifically, level 0 indicates “normal chewing function” and level 4 indicates “no biting and chewing” [17].

### 2.3. Statistical Analysis

Descriptive statistics were performed on both demographic and clinical characteristics in the dataset. Results are presented as mean ± standard deviation or percentage.

## 3. Results

### 3.1. Feeding and Swallowing Evolution from the Neonatal Period to the First Year of Life

Out of the whole cohort, five participants were born pre-term (*n* = 5/14, 36%); among them, two were early pre-term, while the remaining were late pre-term. At birth, all patients (*n* = 14/14, 100%) suffered from immature suckling abilities, requiring enteral feeding to ensure adequate nutrient intake. Full enteral feeding was administered for an average variable period of 4.8 months (range 2–9 months). Exclusive oral feeding was achieved on average at ten months, following an intermediate period of mixed feeding. During the period of transition to entirely oral feeding, compensation strategies were implemented, including modified feeding bottles to favor a greater flow of liquid in cases of significant sucking fatigability or milk thickening to avoid episodes of aspiration due to liquids’ peculiar rheological characteristics. Episodes of dysphagia, characterized by delayed deglutition initiation and poor coordination between sucking, swallowing, and breathing, were observed during the first months of life in eleven patients (78%), together with contraction of the oral–facial musculature and fatigability during sucking. Frequent episodes of regurgitation of bolus from the nasal cavities were reported in two patients (14%). During the first year of life, gastrostomy tube placement was necessary in two patients (14%) and was removed at 18 and 24 months of age, respectively. Furthermore, paroxysmal contractions of the facial and oral–pharyngeal muscles were observed in the whole cohort at birth, especially during crying (*n* = 14/14, 100%), and hypertonia of the neck muscles was present in six (43%) (Supplementary Figure S1).

### 3.2. Feeding and Swallowing Features beyond the First Year of Life

Introduction of solid food was postponed beyond the age of 18 months in almost half of the patients (*n* = 6/14, 43%). Specifically, three patients (21%) were exclusively fed with semi-solid food until they reached the age of 3 years. Two patients (15%) were fed solely with semi-solid food until they reached the age of 5 years.

One patient introduced solid food to his diet at 14 years. Only half of the patients (50%, *n* = 7/14) underwent oral–motor speech training to improve feeding and swallowing skills. In all cases, it was initiated after the first year of life.

During childhood and adolescence, mealtime was characterized by increased duration (>30 min) in six cases (43%), accompanied by fatigue during chewing (43%). Frequent episodes of liquid and solid food spillage from the nasal cavities were reported in three cases (21%) during mealtime. Eight patients reported a poor/reduced appetite (57%). A swallowing disorder was detected in only one case, reaching a total score of 4 when administering the I-Pedi-EAT-10 test. Regarding food consistency, there was no texture restriction in one case (7%). In contrast, in almost all cases (93%), soft solid foods were preferred, while crumbly and crunchy foods were excluded.

**Figure 1:**
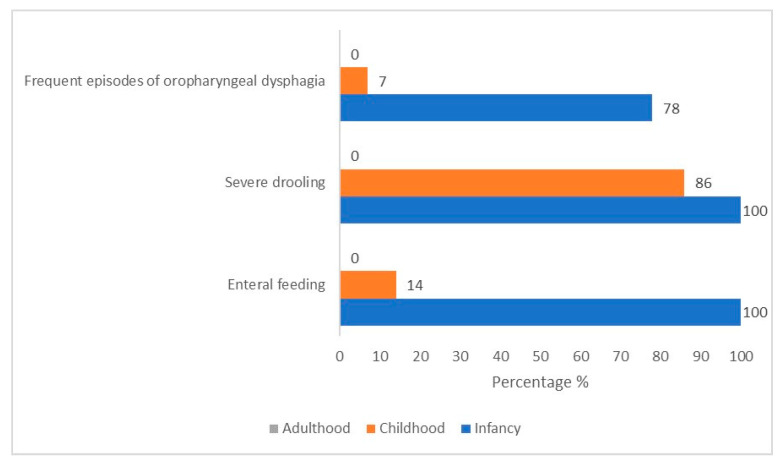
Prevalence variation over time of feeding critical features in our cohort of 14 CS/CISS1 cases.

During childhood, most patients (*n* = 12/14, 86%) presented with mild (only lips wet)-to-profuse (drool off the body and onto objects) sialorrhea, which tended to be especially accentuated with crying. The direct observation of children’s mealtime composed of food of various consistencies revealed a typical chewing pattern (KCPS level of chewing function: 1). Children could chew. However, there were some difficulties in the transition from food to bolus. Specifically, patients could hold, bite, and transfer the solid food with lateral tongue movements to the molar area. Still, there was an inefficacy in breaking down the food between (pre)molar teeth into small pieces with lateral and rotational tongue movements (Figure 1, Supplementary Figure S2, and Table 2).

### 3.3. Feeding and Swallowing Features during Adulthood 

Four adult patients (*n* = 4/6, 67%) reported occasional-to-frequent choking episodes with liquid food bolus (I-EAT-10 mean total score: 1.6; SD: 1.5; total score range: 0–4). Oral–pharyngeal dysphagia in these patients was characterized by a slight delay in the swallowing reflex. In one case, the ENT evaluation revealed the presence of paralysis of the left hemilarynx, with a fixed true vocal cord in a paramedian position associated with restricted breathing space and difficulty in eliciting cough reflex. All but one of the adult patients could prepare and complete meals independently without requiring any supervision or support from caregivers. Drooling saliva was present in all cases, albeit to a milder degree (only lips wet), with occasional occurrence. Direct observation of mealtime revealed prolonged mastication performances. In most patients, the mandible made predominately slight vertical ranges and restricted jaw movements rather than rotary movements (Table 3 and Supplementary Figure S3).

### 3.4. Other Findings

The clinical interview revealed that seven participants (50%) were suffering from gastroesophageal reflux (GERD). Sweets-induced sweating was experienced by nine patients (84%). Multiple untreated carious lesions, in association with lack of oral hygiene, were observed in five patients (36%), including one adult.

In all cases, the direct observation found that language was hypoarticulated; most patients (93%) had a cognitive level within the normal range, while only one (7%) presented a mild impairment.

## 4. Discussion

The present research provides new data on the prevalence of feeding and nutritional issues in patients with CS/CISS1 in a span from birth to adulthood, thus offering a picture of their evolution over the long term.

CS/CISS1 is a series of ultra-rare syndromes exhibiting feeding and swallowing difficulties since birth that vary in degree depending upon neurological, anatomical, and neurodevelopmental issues [18,19].

It is estimated that rare diseases together represent up to 1/10 of all human diseases [20,21]. Extremely rare diseases are sometimes designed as ultra-rare, with a prevalence of <1/50,000 [22]. Given the low prevalence, patients’ data are scarce and scattered and often only single cases are reported in the medical literature [23]. Therefore, one of the main challenges in this field is to collect data from large study cohorts. In the present study, we collected uniform data from a relatively large cohort of CS/CISS1 patients and studied them to improve our knowledge of disease natural history, which is critical for promoting a personalised medicine approach.

The higher risk of serious implications for the respiratory health and the frequent need for hospitalization affecting patients’ quality of life are elements of CS/CISS1 shared with other syndromes, including Down syndrome (DS, OMIM 190685), CTNNB1 syndrome (OMIM #116806), CHARGE syndrome (CS, OMIM #214800), and cardiofaciocutaneous syndrome (CFCS, OMIM#PS115150) [24,25,26,27]. Dysphagia and feeding issue sequelae have also been documented as a source of considerable stress for parents and/or caregivers [28].

In our cohort, in accordance with previous reports [1,7,29], the neonatal period up to the first year of life comprised the most complex period to manage from a nutrition perspective.

Specifically, all our patients presented severe feeding disorders at birth, caused by scarce sucking, poor sucking–deglutition coordination, severe drooling, and facial muscle contractions. At that time, gavage feeding was necessary in order to meet the adequate nutritional intake.

Studies in *Crlf1* and *Clcf1* knockout murine models have shown that mice lacking *CRLF1* or *CLCF1* exhibit sucking deficits, resulting in death within 24 h of birth [30,31]. In the same models, the loss of function of *CRLF1* or *CLCF1* was associated with a reduced number of motor neurons in the facial nucleus [32], which are involved in muscle contraction and relaxation. Accordingly, severe facial muscle contractions and orofacial weakness have been associated to CS/CISS1 [4].

Unlike what has been observed in other syndromes (such as DS, in which structural anomalies and atypical development cause weak lip closure, compression pattern without the use of intraoral suction, and/or dysfunction of upper esophageal sphincter when bottle/breastfed [24]), in newborns with CS/CISS1, the presence of tonic contractions of facial muscles elicited by crying or tactile or painful stimulation can seriously limit the newborn’s ability to feed orally. Paroxysmal episodes of tonic contraction of facial muscles can also be triggered by minimal external stimuli—sometimes hardly detectable—like gastro-esophageal reflux episodes, causing pain [33]. The muscles involved are the mouth and masseter muscles together with orbicular muscles of the eye [34]. During the neonatal period, in CS/CISS1, contractions of the pharyngeal muscles associated with an absent wallowing reflex have also been reported [2]. During these attacks, the vocal cords usually remain in adduction, causing active obstruction and respiratory difficulty [34].

Differently from other genetic syndromes facing severe nutritional issues at birth with a slow recovery of skills over time [27], in CS/CISS1, feeding ability steadily improves in infancy. Only two patients (14%) in our cohort required enteral feeding after the first year of life.

Likewise, in CFCS, long-term enteral feeding dependency is expected, as well as severe food aversion that can persist even beyond adolescence [27].

Interestingly, our study shows that in CS/CISS1, albeit beyond childhood, fully oral feeding was achieved and bolus aspiration was rare in most cases (93%). As previously reported by Hahn and Knappskog, oral motor skills ability, including chewing ability evaluation, is recommended in CS/CISS1 [3]. The presence of limited mouth opening, eventually combined with micrognathia [34], can interfere with optimal chewing. We should note that the presence of multiple untreated caries observed in one third of our cohort might have a deleterious effect on the masticatory performance [35]. In CS/CISS1, the difficulty in maintaining oral hygiene may be also due to motor weakness and camptodactyly of the hands [35].

Moreover, we observed a typical mastication pattern characterized by poor vertical jaw excursion and scarce lateral and rotational tongue movements. Consequently, fatigue and more time-consuming mealtimes were observed in all patients. Similarly to CTNNB1 syndrome [25], a mature chewing pattern does not develop along with growth. In contrast to CTNNB1 syndrome, we did not detect oral–facial and mastication dyspraxia.

Additionally, the peculiar chewing pattern seen in CS/CISS1 was not attributable to the presence of cognitive retardation, particular behavioral patterns, or profoundly poor muscle tone, as observed in Prader–Willi syndrome (OMIM #176270) [36], Smith–Magenis syndrome (OMIM #182290) [37], and Moebius syndrome (OMIM #1579009) [38].

We can speculate a trigeminal involvement in CS/CISS1 considering the chewing act’s poor amplitude and intensity. Moreover, the presence of trismus observed in CS/CISS1 is commonly associated with signs and symptoms of trigeminal sensory and motor dysfunction, including corneal alterations [36] and dysfunction of the palatine veil, causing nasal regurgitation and excessive passage of the air from the nose during phonation [39]. This specific chewing pattern remains through adulthood.

## 5. Conclusions

The challenges encountered by affected patients and their families concern not only diagnosis but also the possibility of receiving timely and appropriate disease-specific treatments. Feeding difficulties are a constant feature of patients with CS/CISS1, and here we report their prevalence and evolution from birth to adulthood. By characterizing and expanding the phenotype associated with CS/CISS1, our experience might improve the patients’ management of the condition and support the introduction of preventive habits with respect to this ultra-rare disease.

## 6. Limits and Future Research

In light of the complexities associated with rare diseases and the limited number of cases, advancing our comprehension of feeding and swallowing proficiency necessitates ongoing research and interdisciplinary partnerships to create and validate both consistent and replicable assessments for future characterization and measurement. A genotype/phenotype correlation based on the type or localization of *CRLF1* variants cannot yet be established. Further research and collaborations are warranted to advance our knowledge of the genotype–phenotype correlations.

## Figures and Tables

**Table 1:** Details of our cohort of patients with CS/CISS1.

Participants (*n =* 14)	
Demographics at the time of our study	
Age range (years)	6–44
Median age (years), SD age (years)	18 ± 10.62
Gender (Male)	6
Genetics *n* (%)	
*CRLF1*	14 (100)
Signs and symptoms (%)	
Hyperhidrosis	14 (100)
Camptodactyly	13 (93)
Chubby cheeks	13 (93)
Hyperthermia	13 (93)
Anteverted nostrils	11 (79)
Scoliosis	11 (79)
High arched palate	9 (64)
Depressed nasal bridge	8 (57)
Micrognathia	7 (50)
Hypertonia	6 (43)

SD: standard deviation.

**Table 2:** Prevalence variation over time of feeding critical features in our cohort of 14 CS/CISS1 cases.

Key Features	Prevalence (%)		
	Infancy	Childhood	Adulthood
Enteral feeding	100	14	0
Mild-to-severe drooling	100	86	0
Severe oral–pharyngeal dysphagia	78	7	0

**Table 3:** Nutritional abilities and genetic variants in our cohort of 14 patients with CS/CISS1. Y: year; M: male; F: female; na: not applicable; +: present; −: absent; H: homozygosity; CH: compound heterozygosity.

Patient ID		1	2	3	4	5	6	7	8	9	10	11	12	13	14	Prevalence (%)
Age (y)		6	6	9	10	11	11	13	17	19	21	25	30	30	44	
Gender		F	M	M	M	M	F	F	F	F	F	M	F	M	F	
Infancy	Poor suck at birth	+	+	+	+	+	+	+	+	+	+	+	+	+	+	100
	Enteral feeding at birth	+	+	+	+	+	+	+	+	+	+	+	+	+	+	100
	Contraction of facial muscles	+	+	+	+	+	+	+	+	+	+	+	+	+	+	100
	Gastrostomy feeds	−	+	−	−	−	−	+	−	−	−	−	−	−	−	14
	Severe drooling	+	+	+	+	+	+	+	+	+	+	+	+	+	+	100
	Oral–pharyngeal dysphagia	−	−	+	+	+	+	+	−	+	+	+	+	+	+	78
	Hypertonia of neck muscles	−	+	−	−	−	−	−	+	+	+	−	+	−	+	43
	Nasal regurgitation	−	−	−	+	−	−	−	−	−	+	−	−	−	−	14
Childhood	Impaired mastication	+	+	+	+	+	+	+	+	+	+	+	+	+	+	100
	Preference for soft solid food textures	+	+	+	+	+	+	+	+	+	+	+	+	+	−	93
	Drooling	+	+		+	+	+	+	+	+	+	+	+	+	−	86
	Poor appetite	+	−	+	+	+	−	+	−	+	−	+	−	+	−	57
Adulthood	Occasional drooling	na	na	na	na	na	na	na	na	+	+	+	+	+	+	100
	Occasional oral–pharyngeal dysphagia	na	na	na	na	na	na	na	na	+	+	−	−	+	+	67
Gene	*CLRF1*	+	+	+	+	+	+	+	+	+	+	+	+	+	+	100
Variant		c.676_677insA	c.(697+167_?) del; chr 19 g. (18695989_18740580) del	c.676_677insA	c.676_677insA	c.221T>C; c.676_677insA	c.676_677insA	c.226T>G; c.676_677insA	c.[338 A>T;341 T>C]	c.226T>G	c.23-45dup	c.676_677insA	c.226T>G; c.676_677insA	c.935G>A; c.856-? c.1269+?del	c.226T>G; c.676_677insA	
Zygosity		H	CH	H	H	CH	H	CH	H	H	H	H	CH	CH	CH	
Exon(s) involved		4	5–9; 1–9	4	4	2; 4	4	2; 4	2	2	1	4	2; 4	6; 6–9	2; 4	

## Data Availability

The datasets generated during the current study are available from the corresponding author upon reasonable request.

## Supplementary Materials

The following supporting information can be downloaded at https://www.mdpi.com/article/10.3390/genes15091109/s1: Figure S1. Prevalence of of feeding critical features during infancy. Figure S2. Prevalence of of feeding critical features during childhood. Figure S3. Prevalence of of feeding critical features during adulthood.

## Abbreviations

CS/CISS	Crisponi/cold-induced sweating syndrome
I-EAT-10	Italian version of the Eating Assessment Tool-10
I-Pedi-EAT-10	Italian version of the Pediatric Eating Assessment Tool
KCPS	Karaduman Chewing Performance Scale
