# Supplementary material for: Feeding and Nutritional Key Features of Crisponi/Cold-Induced Sweating Syndrome
Source: Genes (Basel). 2024 Aug 23;15(9):1109. doi: 10.3390/genes15091109 (PMC11431494; doi:10.3390/genes15091109)

Supplementary Figure S1. Prevalence of of feeding critical features during infancy.

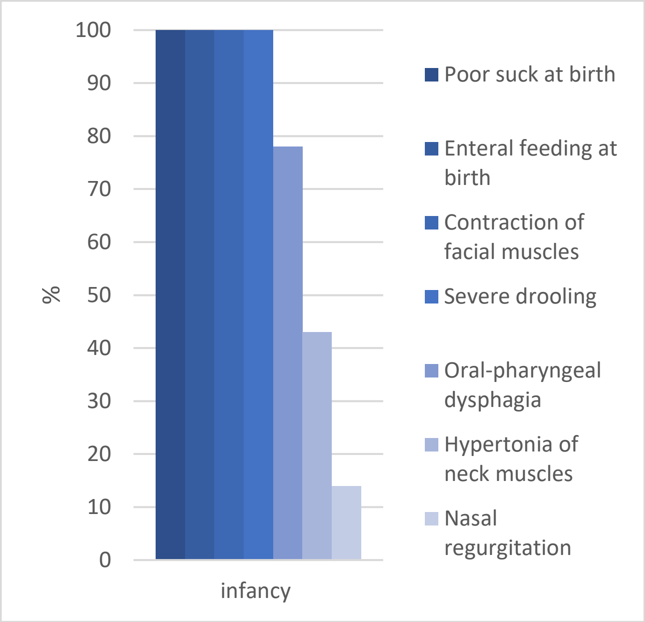

Supplementary Figure S2. Prevalence of of feeding critical features during childhood.

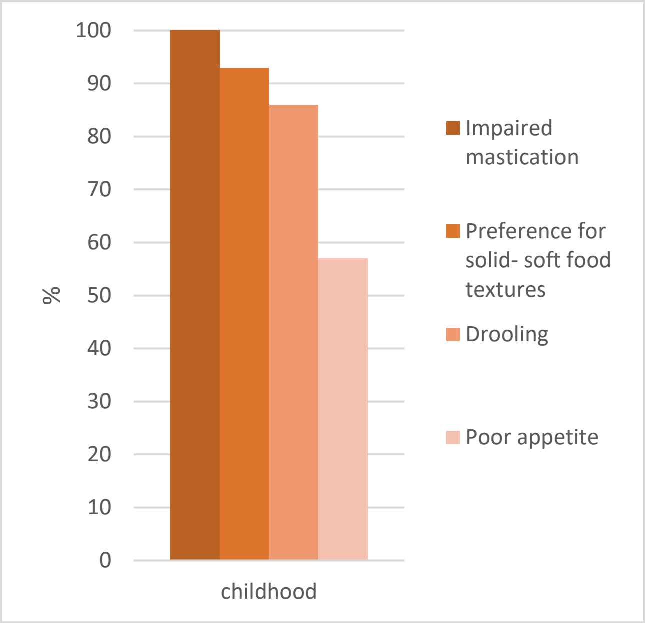

Supplementary Figure S3. Prevalence of of feeding critical features during adulthood.

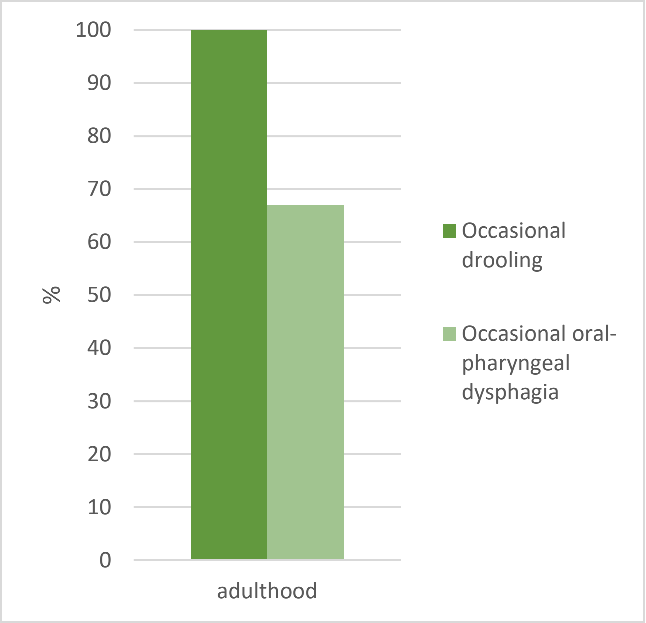

Supplement: Supplementary file 1 [file genes-15-01109-s001.zip › genes-3110552-supplementary.pdf]
